# *Nigella sativa*: A Comprehensive Review of Its Therapeutic Potential, Pharmacological Properties, and Clinical Applications

**DOI:** 10.3390/ijms252413410

**Published:** 2024-12-14

**Authors:** Adina Alberts, Elena-Theodora Moldoveanu, Adelina-Gabriela Niculescu, Alexandru Mihai Grumezescu

**Affiliations:** 1 Carol Davila University of Medicine and Pharmacy, 050474 Bucharest, Romania; adina-magdalena.alberts@rez.umfcd.ro; 2 Department of Science and Engineering of Oxide Materials and Nanomaterials, National University of Science and Technology Politehnica Bucharest, 011061 Bucharest, Romania; moldoveanu.theodora99@gmail.com (E.-T.M.); adelina.niculescu@upb.ro (A.-G.N.); 3 Research Institute of the University of Bucharest—ICUB, University of Bucharest, 050657 Bucharest, Romania

**Keywords:** *Nigella sativa*, thymoquinone, thymol, antioxidant, anti-inflammatory effect, antimicrobial, immunomodulator, analgesic, antipyretic

## Abstract

*Nigella sativa* (NS) is an annual herb belonging to the Ranunculaceae family, also known as black cumin or black seed. This plant has been used since ancient times due to its therapeutic properties and has proven effective in gastrointestinal, respiratory, cardiovascular, infectious, and inflammatory conditions. In this review, the aim is to highlight the therapeutic effects of the plant known in Arab countries as “the plant that cures any disease”, which are provided by the phytochemical compounds in its composition, such as thymoquinone, p-cymene, α-thujene, longifolene, β-pinene, α-pinene, and carvacrol. These compounds confer an antioxidant effect to the seeds, leading to a significant decrease in ROS and a potent anti-inflammatory effect. Also, in this review, the aim is to highlight that NS seeds may have a synergistic effect with other drugs, such as chemotherapeutic agents or antibiotics, which may lead to a reduction in the therapeutic dose, may have an improved effect, and could lead to overcoming obstacles such as drug resistance. The studies provided in this review showed that NS has the potential to be a therapeutic agent both as a monotherapy and as an adjuvant. Although there are studies demonstrating the therapeutic properties of NS, there is a need for much more extensive research and more clinical trials with clearly established objectives so that the mechanism of action of the active substances in NS is much better understood. With the data provided so far, NS can be used in food and drug production in small quantities and can be administered for short periods. Further investigations may lead to an understanding of the therapeutic profile and the most effective mode of administration, as well as a clearer perspective on the toxicological profile of NS.

## 1. Introduction

**Figure 1:**
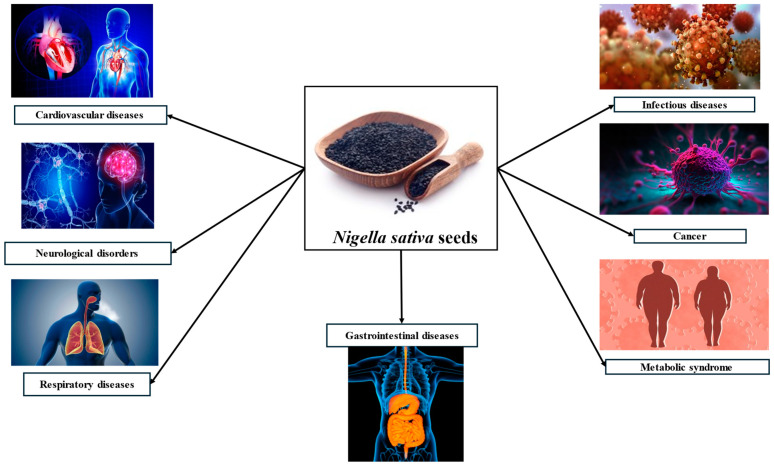
Therapeutic applications of *Nigella sativa*. Created based on information from [3].

*Nigella sativa* (NS) is a plant that grows to a height of 20–30 cm and has linear leaves, with flowers colored light blue and white, which form a fruit in the form of a large, swollen capsule with three to seven connected follicles containing the seeds. They are often used as a spice in gastronomy and as a herbal treatment. NS is native to most countries in the Eastern Mediterranean, North Africa, South-East Asia, and the Indian subcontinent, and also has a long history of cultivation in the Ethiopian region. NS can be cultivated in many countries, but it is predominantly found in Greece, Turkey, Egypt, Iran, Syria, Albania, Saudi Arabia, India, and Pakistan. Also, Malaysia is the largest exporter of NS essential oil, followed by Indonesia, China, the Netherlands, Germany, Sweden, India, the United States, Russia, and Spain [1,2,3,4]. This plant is well-known for its beneficial impact on human health; as a result, traditional medicine frequently uses the plant’s seeds to treat aches, diarrhea, rheumatism, back pain, and other conditions such as hypertension, diabetes, respiratory diseases, gastrointestinal affections, cancer, and migraine. It also functions as an antibacterial and anti-inflammatory agent, and NS is an effective diuretic. Thymoquinone (TQ) is the most important compound in NS, along with additional active substances such as p-cymene, α-thujene, longifolene, β-pinene, α-pinene, and carvacrol. NS is used in medicine in various forms, including essential oils, powders, pills, and extracts of these seeds [3,5,6,7,8]. Researchers’ interest in NS seeds has expanded because they have also demonstrated various health benefits. It has been demonstrated that the primary ingredient, TQ, improves the reduction in oxidative stress and reactive oxygen species (ROS) production, lowers inflammation, boosts immunity, and supports energy metabolism and cell survival. Additionally, it might offer a defense against major illnesses (Figure 1), like cancer and neurological diseases, as well as against metabolic, digestive, hepatic, cardiovascular, renal, and respiratory conditions [3].

NS was first mentioned in the scientific literature about a century ago and is also mentioned in numerous historical (e.g., Hippocrates and Dioscorides) and religious texts (e.g., Isaiah–the Old Testament, Easton’s Bible Dictionary) of several civilizations, including Egyptian, Ayurveda, Siddha, Unani, Greco-Roman, Malay, and Jewish [9,10]. As such, NS has been used since ancient times in traditional folk medicine worldwide to treat all types of ailments and diseases. NS seeds were used to treat psychiatric, lung, kidney, stomach, and liver ailments [3,9,10,11]. In the modern era, NS has been noticed and studied for its properties, and significant advances in its research have been made in the last twenty years. Although studies are being carried out worldwide, most of the research on NS has been carried out in the Middle East and South Asia. In this way, the pharmacological and biochemical aspects are highlighted, which provides perspectives for using this natural product in clinical medicine. So, NS has been noted as a potential anti-inflammatory and antioxidant agent, and a protector against cancer and other diseases such as diabetes, heart problems, neurological diseases, etc. [3,10,11,12].

Thus, this review aims to comprehensively explore NS’s role in health preservation, disease prevention, and treatment. Even though the subject was previously discussed in some publications [3,13,14,15], these works have included studies and research conducted until 2020–2021, mainly emphasizing the potential of thymoquinone as a biologically active substance in the therapeutic effects of NS. However, the present work first provides a scientific basis by discussing the main compounds of NS with their therapeutic effects, molecular structure, properties, and mechanisms of action. Then, the applications of NS in the prevention and treatment of various diseases are analyzed in detail, evidencing the therapeutic potential of these seeds. In this review, areas of ongoing discussion are also acknowledged. The need for further research is pointed out to consolidate its role in clinical practice, improve patient’s quality of life, and discover new therapeutic opportunities. In this regard, papers published in English between 2019 and 2024 (e.g., reviews, meta-analyses, preclinical and clinical studies) were selected and analyzed in this review. The information was retrieved from scientific databases such as Google Scholar, PubMed, MDPI, Science Direct, Scopus, Web of Science, SpringerLink, and Wiley Online Library, using various combinations between the following keywords: “*Nigella sativa*”, “Black Cumin”, “thymoquinone”, ”bioactive compounds”, “pharmacological properties”, “antioxidant”, “anti-inflammatory”, “antimicrobial, cancer”, “metabolic disease”, “neuroprotective”, and “clinical trials”.

## 2. Phytochemical Composition

NS has had a significant role in various culinary, gastronomic, and medical activities throughout the populations of the Mediterranean, Europe, South Asia, and the Middle East. Antioxidant, anti-inflammatory, analgesic, antiviral, antihistamine, antidiabetic, diuretic, hypotensive, hypolipidemic, immunomodulatory, hepatoprotective, neuroprotective, nephroprotective, and anticarcinogenic are just a few of the amazing properties of these seeds. With their flavor and bitterness, NS seeds are great as a spice or in addition to bread, pickles, tea, coffee, and salads, and they can be combined with honey. Additionally, NS can be a great source of high-quality oils and proteins with positive health effects. They can serve as a dietary source to fulfill daily needs for protein and minerals [16,17,18]. NS is known for the multitude of bioactive compounds reported in the literature (Table 1).

**Figure 2:**
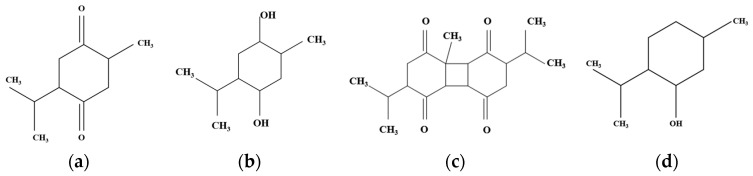
Chemical structure of the most important NS compounds: (**a**) thymoquinone (TQ); (**b**) thymohydroquinone (THQ); (**c**) dithymoquinone (DTQ); (**d**) thymol (THY). Created based on information from [56].

NS contains both volatile and non-volatile oils, proteins, carbohydrates, minerals (e.g., iron, calcium, potassium, magnesium, zinc, and copper), vitamins A and C, as well as phytochemicals such as sterol (e.g., β-sitosterol, campesterol, stigmasterol, and 5-avenasterol) and saponins, phenolic compounds, alkaloids, lipid constituents, and fatty acids (e.g., linoleic, linolenic, oleic, palmitoleic, palmitic, arachidonic, stearic acids). More than forty different compounds (e.g., trans-anethole, p-cymene limonene, carvone, α-thujene, α-thujene, thymoquinone (TQ) (Figure 2a), thymohydroquinone (THQ) (Figure 2b), dithymoquinone (DTQ) (Figure 2c), thymol (THY) (Figure 2d), carvacrol, and β-pinene) with varying concentrations have been identified in the composition of NS essential oils. TQ is the most important compound in these seeds [4,51,52,53]. NS seed oil can be obtained using supercritical carbon dioxide at temperatures of 40 °C and pressures varying between 10 and 35 MPa. It was observed that at lower pressures (10–15 MPa), thymoquinone-rich fractions, ideal for health applications, were obtained, while at higher pressures (35 MPa), the extraction focused on polyunsaturated fatty acids suitable for the food industry [54]. An environmentally friendly solvent, 2-methyltetrahydrofuran (MeTHF), can be used to replace hexane in the extraction of bioactive lipids from NS seeds. Extraction with MeTHF was shown to produce a higher yield (34%) than the conventional hexane method (29%). The oil obtained from NS was rich in linoleic acid (61%) and oleic acid (19%), and MeTHF enhanced the extraction of major phenolic compounds such as thymol [55].

TQ (C_10_H_12_O_2_) is one of the most important active compounds in the composition of NS seeds, having a molecular weight of 164.204 g/mol and found in essential oils in a concentration of 18–25 µg/mL [57,58]. This compound is recognized for its potent hepatoprotective effects, antioxidants, anti-inflammatory anticarcinogenic, and pro- and anti-apoptotic properties. TQ belongs to the class of monoterpene compounds formed by the condensation of two isoprene units. Secondary metabolism in plants is responsible for forming these compounds, which are isolated by steam distillation or solvent extraction of plant parts. Numerous methods exist to isolate and purify TQ, including supercritical fluid extraction, hydrodistillation, soxhlation, and chromatographic techniques. A comparison of the methods showed that Soxhlet extraction yields TQ in yields up to 48%, while hydrodistillation yields only 3%. TQ was also purified to 97% purity by high-performance liquid chromatography (HPLC). TQ is a hydrophobic compound that is found in tautomer form. This limits its bioavailability, although this is high. When administered orally, absorption is slow and elimination much more rapid, whereas intraperitoneal injection at high concentrations may produce toxicity. The dose must be low (100 mg/kg) for oral administration to avoid toxic effects. Also, TQ can be sensitive to external factors such as light and is unstable in alkaline media, which prevents its applicability in the pharmaceutical field. To mitigate these drawbacks and to increase bioavailability, formulations based on nanocarriers (e.g., liposomes or lipid nanoparticles) are proposed [58,59,60,61]. Thymohydroquinone (THQ) and dithymoquinone (DTQ) are two compounds chemically derived from TQ. THQ, also known as DTQ or hydrothymoquinone, is the phenolic hydroquinone derivative of thymoquinone; THQ represents the first reducing product of TQ, which is a significant co-product of the essential oil of NS. Several investigations on the biological activity of TQ are covered in the literature. These include its effects on hypertension, hepatoprotection, antitumor proliferation, anti-inflammatory properties, and protection against neuropathy, as well as gastroenterological, renal, and cardiac disorders. The use of DTQ or THQ is not concerning, in contrast to thymoquinone [23,62]. Thymol (C_10_H_14_O) is a naturally occurring, colorless, crystalline monoterpene phenol with a ten-carbon unit, also known as 2-isopropyl-5-methylphenol. It is highly soluble in alkaline solutions, alcohols, and other organic solvents but is sparingly soluble in water at a neutral pH. It is present in NS seeds and thyme oil and is used as an active substance in various applications, including food, pharmaceuticals, cosmetics, etc. THY has been shown to have excellent anti-inflammatory (inhibits the production of cytokines and chemokines), antioxidant, and antihyperlipidemic properties [63]. Thymol can be extracted using solvent-liquefied dimethyl ether (DME), being an alternative to traditional solvents (e.g., hexane, ethanol, etheric petroleum), which may have drawbacks such as high toxicity and low efficiency. By using DME, a much better yield (21%) has been demonstrated under low-temperature conditions using ground black cumin seeds. This method is simple, cost-effective, and easy to apply and allows recovery of the DME solvent for reuse [64]. Another compound found in NS essential oil is represented by carvacrol, also known as 2-methyl-5-(1-methylethyl) phenol. It is well known for its anti-inflammatory, antioxidant, antitumoral, analgesic, and antihepatotoxic properties and its antimicrobial effect by damaging the bacterial membrane. P-cymene, also known as 1-methyl-4-(1-methylethyl)-benzene, represents the biological precursor of carvacrol, and it is less effective than carvacrol used alone [65].

## 3. Pharmacological Properties

**Figure 3:**
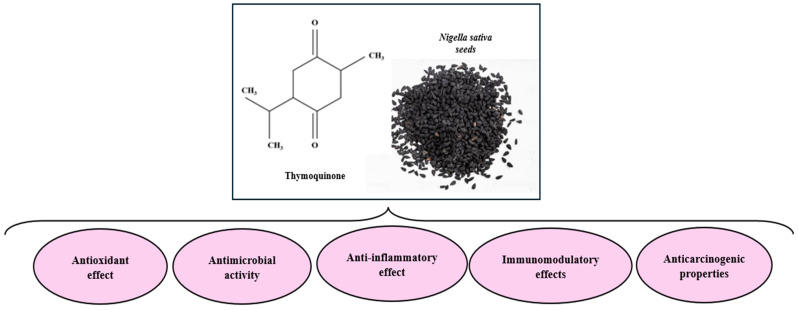
Pharmacological Properties of *Nigella sativa*. Created based on information from [67].

It has been shown that NS oil exhibits excellent anti-inflammatory, antioxidant, immunomodulatory, and anticarcinogenic properties. The beneficial effects on glucose homeostasis and lipid profile have also been studied, as well as the fact that it may have gastrointestinal and hepatic protective, antitussive, and cardioprotective effects, including antihypertensive effects [66]. These beneficial effects of NS seeds have been attributed to the high TQ content they have (0.05–6.19 mg/mL) [67]. The main pharmacological properties of NS are illustrated in Figure 3 and will be discussed in the following subsections.

### 3.1. Antioxidant and Anti-Inflammatory Effects

TQ activity is closely linked to its redox capacity, which leads to free radical scavenging and modulation of endogenous antioxidant systems. For example, it was observed that TQ led to the upregulation of antioxidant enzymes such as catalase (CAT), glutathione peroxidase, and superoxide dismutase (SOD), enhancing effects on oxidative stress, leading to hepatoprotective effect, blocked Nuclear factor erythroid 2-related factor 2 (Nrf2)/Heme oxygenase-1 (HO-1) pathway, which led to the protection of epithelial cells from oxidative stress and effects on rheumatoid arthritis in experimental models. TQ reduces oxidative stress by reducing ROS and decreasing levels of IL-1β, IL-6, TNF-α, and IFN-γ. Thus, its beneficial effects have been attributed to improving general well-being in patients suffering from cardiovascular disease, neuroinflammation, and cancer [67]. TQ may exhibit an anti-inflammatory effect due to its antioxidant activity and ability to inhibit oxidative stress. An example may be represented in the amelioration of experimental autoimmune encephalomyelitis (EAE), which may be attributable to multiple sclerosis (MS), in which TQ had a prevalence efficacy of 90% and a therapeutic efficacy of 50%. TQ increased the expression of glutathione (GSH) levels and prevented oxidative stress damage, significantly reducing the symptoms of EAE [66]. TQ also demonstrated inhibitory activity against the expression of malondialdehyde (a product of lipid peroxidation) [68]. It was observed that TQ can reduce the activity of the inflammatory factors COX-2, MAPK, and NF-κB. TQ has also been shown to reduce inflammation and oxidative stress in cardiovascular disease, improve cardiac function, and reduce infarct size by inducing autophagy and reducing apoptosis. In addition, TQ reduced the occurrence of cardiac markers LDH and CK-MB, which implicitly led to a decrease in oxidative stress and inflammation. In neurodegenerative diseases (e.g., Alzheimer’s (AD), Parkinson’s (PD), Huntington’s (HD)) TQ demonstrated beneficial effects. In PD, TQ has been shown to reduce the activity of superoxide dismutase and catalase, inhibiting the expression of COX-2 and iNOS, which produce inflammation, and beta-amyloid, which is responsible for the development of PD. In AD, TQ demonstrated the ability to reduce inflammation and the neurotoxicity of LPS and IFN-γ activated cells by significantly inhibiting the NF-κB signaling pathway, leading to the protection of neurons against damage. TQ was also shown to reduce levels of AD-associated inflammatory factors, TLR2, TLR4, TNF-α, and IL-1β. In HD, it was observed that TQ significantly reduced the expression of glial fibrillary acidic protein (GFAP) and pro-inflammatory cytokines, thus leading to improved motor function and reduced neuronal inflammation [66].

### 3.2. Antimicrobial Activity

Infections with drug-resistant microorganisms (MDRs) represent a major public health problem, as pathogens can rapidly acquire drug resistance. TQ has demonstrated that it can have a substantial antimicrobial effect against several pathogens, especially Gram-positive bacteria (e.g., *Staphylococcus aureus*), but less effective against Gram-negative strains. A study conducted by Fatima Mustafa Al-Najar [69] focused on demonstrating the antimicrobial effect of NS against Gram-positive (*S. aureus*) and Gram-negative (*E. coli*) strains involved in urinary tract infections. Thus, the results show that the alcoholic extract of NS had an inhibitory effect on bacterial growth. It was also noted that NS has a good effect on both strains and that it could be used to obtain therapeutic agents to treat urinary tract infections. This may decrease the antibiotic resistance that occurs in bacteria. Another study conducted by Sakar Ahmed Abdullah et al. [70] analyzed the inhibitory effect of NS oil, especially on Gram-positive strains. It was also observed that NS oil also had an inhibitory effect on multi-drug-resistant bacteria (MRSA), thus proving that NS could be an effective strategy against bacterial skin infections. Another study was conducted by Aalaa A. Chmagh et al. [71], which focused on investigating the antimicrobial and antibiofilm potential of NS extract. The species on which the tests were performed were as follows: *Bacillus cereus*, *Escherichia coli*, *Klebsiella pneumoniae*, *Staphylococus aureus*, and the NS extracts were made with different solvents (water, methanol, ethyl acetate, hexane). The results obtained show that the methanol extracts had the highest antimicrobial activity on *B. cereus* and *K pneumoniae*, demonstrated by measuring the inhibition diameter. The minimum inhibitory concentration showed a moderate to high effect against *S. aureus* and *K. pneumoniae* strains. In terms of biofilm formation, the extracts inhibited *S. aureus* by about 77%.

The antimicrobial activity of TQ has been tested using silico assays, but these require further validation. Also, TQ has demonstrated an anti-biofilm potential against many pathogens in an optimal timeframe tested by time-kill assays. Kamal A. Qureshi et al. [72] chose to compare the results obtained with other studies, where TQ showed strong activity against Gram-positive bacteria, where the minimum inhibitory concentration ranged from 3 to 6 µg/mL, while for Gram-negative bacteria, 200 to 1600 µg/mL was required, and TQ was not effective against *Escherichia coli* and *Pseudomonas aeruginosa*. Regarding SARS-CoV-2 infection, TQ effectively prevents COVID-19-induced oxidative stress due to its potent antioxidant properties. Thus, NS reduced inflammation and lung damage induced by the infection through the direct interaction of NS compounds with SARS-CoV-2 spike proteins, preventing the virus from entering the cells. TQ also prevented the formation of advanced glycation end-products (AGEs), which often lead to severe complications in diseases such as diabetes and following COVID-19 infection. Thus, TQ considerably reduced albumin glycation and protected erythrocytes against hemolysis by stabilizing their membrane [73]. In animal tests, NS seed extract significantly increased the subjects’ immunity, conferring high protection against infections caused by different strains. At the same time, the extract improved intestinal barrier integrity by increasing the expression of tight junction-associated genes and reduced the levels of pro-inflammatory cytokines (e.g., IL-1β, IL-6, and TNF-α) and by increasing the anti-inflammatory cytokine IL-10. Together with other antimicrobial agents, NS seed extract led to an improved effect against methicillin-resistant *Staphylococcus aureus* strains. Thus, the compounds in the NS extract caused changes in the cell wall morphology of the bacteria and produced microscopically detectable structural changes, which proves its efficacy in combating pathogens [74]. Because of its antimicrobial activity and anti-inflammatory and antioxidant properties, NS is an appealing possibility for managing infections caused by microorganism pathogens that are resistant to drugs and for use in other therapeutic applications, such as managing and preventing COVID-19 and its complications.

In Section 2, the bioactive substances of NS and their biological effects are presented. Thus, as far as NS’s antimicrobial potential, thymoquinone is not the only bioactive substance that fulfills this role. In a study conducted by Ahmad Mouwakeh et al. [75], it was demonstrated that thymoquinone, carvacrol, and p-cymene had antimicrobial potential against the *L. monocytogenes* strain by considerably decreasing the minimum inhibitory concentration of erythromycin and ethidium bromide (EtBr). A review of several studies performed by Anna Marchese et al. [76] mentions that the use of p-cymene may be a promising alternative for use in polymeric materials or nanomaterials due to its antibacterial and anti-inflammatory effects. An in silico study performed by Athanasios Panagiotopoulos et al. [77] demonstrates that p-cymene interacts with the binding domain of N SARS-CoV-2, known as Importin, and thus prevents the association with the IMPα-IMPβ-RanGDP complex. It was observed that p-cymene had an antiviral effect at low concentrations in vitro for both SARS-CoV-2 and other RNA viruses. However, against influenza A, it was observed that p-cymene did not completely cure the infection but could be combined with other antivirals for improved effects. The researchers conclude that in vitro and in vivo studies are needed to confirm the efficacy and safety of this substance.

### 3.3. Immunomodulatory Effects

NS is also known for its immunomodulatory effects, having the ability to influence and regulate the immune system responses. Thus, in vitro, in vivo, and clinical studies have highlighted the immunomodulatory activity of this plant, in particular, the main active compound TQ. In this way, a study [78] performed on human lymphocytes and polymorphonuclear leukocytes led to the following results. A high concentration of NS extract led to suppressing the lymphocyte response to all mitogens by increasing cell death while suppressing the phagocytic activity of polymorphonuclear leukocytes. It was also observed that the NS extract led to the synthesis of interleukins IL-1β and IL-3 but not IL-2. Another study [79], performed on C57/BL6 and BALB/c cell lines, demonstrated that the use of aqueous NS extract led to cell proliferation, Th2 cytokine secretion, and inhibition of the secretion of pro-inflammatory mediators TNF-α, IL-6, and nitric oxide (NO) by macrophages. As an animal model of asthma, Boskabady and colleagues [80] investigated the immunomodulatory effects of an ethanolic extract of NS seed in ovalbumin (OVA)-sensitized guinea pigs. The researchers demonstrated that NS extract significantly decreased pathogenic alterations in the subject’s lungs. Localized epithelial necrosis and the infiltration of lymphocytes and eosinophils were diminished. When NS extract was administered to rats, blood levels of IFN-γ and IL-4 increased concurrently in comparison to control animals. These findings supported the NS extract’s ability to reduce pulmonary inflammation. Because TQ suppresses NF-κB, MAPK, PPAR-γ, and STAT, it may be useful in the treatment of allergy symptoms. TQ has been shown in in vivo studies to drastically lower eosinophil infiltration of the nasal mucosa and inflammatory markers like IgE, IL-1β, IL-4, and TNF-α in experimental models of allergic rhinitis and asthma. *N. sativa* oil improved symptom control and decreased blood eosinophilia in children and adults with asthma, and it also reduced the symptoms of allergic rhinitis in clinical investigations. Additionally, supplementation with NS oil reduced inflammation and lung function in asthmatic patients, and safety studies indicate that it may be taken regularly without causing noticeable side effects while lowering triglycerides and total cholesterol [81].

A double-blind, randomized, placebo-controlled clinical trial by Ayad Salem et al. [82] was conducted on 52 volunteers aged 18–25 years over a 4-week period. The control group took activated charcoal capsules (placebo), while the other group received 0.5 g, 1 g, or 2 g of NS powder in capsules. Blood samples of the volunteers were taken before and after treatment to observe liver and kidney function, hematological parameters, and cytokines (IL-1, IL-4, IL-6, IL-10, TNF). A subgroup was also tested for immunoglobulins and expression of genes involved in the immune response. In terms of immune response, NS led to a significant increase in li lymphocytes, as well as CD^3+^ T cells and CD^4+^ T cells. It was also observed that this effect was dose-dependent, and at high doses of 2 g, this effect was absent. Also, no effects on immunoglobulins or cytokines were recorded. Other beneficial effects were also recorded. In the group receiving 0.5 g NS, there was a significant decrease in systolic blood pressure and heart rate; in the group receiving 1 g, there was a significant reduction in diastolic blood pressure. Another effect, seen in patients who received 1 g or 2 g NS, was reported to be increased energy levels and improved sleep.

The immunomodulatory effects of NS may also be a key factor in cancer treatment. Thus, several studies have investigated the active substance in the composition of NS seeds, namely TQ, in this respect. Thus, one of the studies [83] focused on investigating the inhibitory effect on the epigenetic code of cancer cells, which is controlled by the UHRF1/DNMT1/HDAC1 complex. UHRF1 is present in many tumors and has been observed to be a factor that promotes cell proliferation, inhibits apoptosis, and also represses tumor suppressor genes. By administration of TQ, it was observed that this active substance has the potential to degrade the UHRF1 protein by interacting with its RING domain. It can also interact with the SRA and DNMT1 domains by inhibiting their activation, thus inducing global DNA hypomethylation. Also, TQ has demonstrated a cytotoxic effect in vitro on leukemic cells by inhibiting DNMT1 and HDAC1, representing a potential epigenetic treatment against cancer [83].

In HL60 leukemic cells, c-Myc is overexpressed and thus associated with chemotherapy resistance. Thus, TQ administration led to the inhibition of the JAK/STAT and PI3 K/AKT/mTOR signaling pathways, which stimulate c-Myc expression and are associated with the inhibition of proliferation and induction of apoptosis in cancer cells [84]. Another study [84] highlighted the potential of TQ to inhibit PI3K/Akt/mTOR and JAK/STAT pathways in cancer cells. Thus, TQ was tested on MV4–11—B-myelomonocytic leukemia (AML) and K562 (CML) chronic myelogenous leukemia cells, on which it had a dose- and time-dependent inhibitory effect, with an efficiency of 99% after 72 h. TQ significantly decreased mRNA levels for PI3K, Akt, and mTOR and increased PTEN gene expression in both cell lines, with similar effects in previous studies on other cancers. TQ has also been shown to reduce the activation of FLT3 and BCR-ABL, two oncoprotein proteins involved in AML and CML leukemogenesis [84].

## 4. Therapeutic Applications

NS seeds have long been used to cure various diseases and ailments. Islamic literature considers it one of the most efficacious forms of medical therapy. Its frequent usage in Tibb-e-Nabwi (Prophetic Medicine) has been recommended. It is frequently used to treat skin conditions, digestive issues, anti-diarrhea, liver tonic, analgesics, and hypertension conditions. Many researchers have conducted extensive studies on a wide range of NS’s pharmacological actions, which may include antidiabetic, anticancer, immunomodulator, analgesic, antimicrobial, anti-inflammatory, spasmolytic, bronchodilator, hepato-protective, renal protective, gastro-protective, antioxidant properties, etc. [85].

### 4.1. Role in Metabolic Disorders

One of the most common metabolic diseases affecting people of all ages, genders, and races is diabetes mellitus (DM). Because of their combined effects, medicinal herbs have attracted a lot of interest from researchers and are thought to be a good adjuvant agent to oral antidiabetic medications. Type 2 diabetes mellitus (T2DM), which results from the interplay of environmental factors and genetic susceptibility, accounts for 90% of instances of DM. One characteristic of T2DM is insufficiency in the manufacturing of insulin, which causes blood glucose levels to rise. Reactive oxygen species (ROS), which cause cell damage and accelerate the onset of diabetic complications such as autonomic neuropathy, nephropathy, and diabetic retinopathy, are encouraged by this rise [86].

In this context, NS has been studied for treating or relieving DM-specific symptoms. Thus, an analysis was performed that highlighted the effects of NS administration together with other oral antidiabetic drugs on several glycemic parameters (e.g., fasting blood glucose (FBG), 2 h postprandial glycemia (2hPG), glycosylated hemoglobin (HbA1c), insulin levels, and insulin resistance). It was observed that the administration of NS significantly reduced glycemia in streptozotocin-treated diabetic mouse models, and HbA1c levels were also reduced in TQ-treated diabetic rats. Also, NS administration led to an improvement in pancreatic β-cells and thus reduced insulin resistance. The high polyphenol content of NS helps repair pancreatic β-cells, which in turn enhances the metabolism of fats and carbohydrates in hyperglycemic circumstances. This may be due to NS’s ability to activate the insulin and adenosine monophosphate-activated protein kinase (AMPK) signaling pathways. Additionally, NS may increase the expression of PI3K kinase, GLUT-4, and insulin-like growth factor-1 (IGF-1) to enhance the transport and utilization of glucose. Furthermore, NS is effective in blocking sodium–glucose co-transporters, preventing intestinal glucose absorption, and may also reduce the oxidation of cholesterol and triglycerol lipoproteins, which may lead to a significant improvement in hyperlipidemia in diabetics. Another major factor is the antioxidant potential of NS. In DM, elevated glucose levels can induce oxidative stress and thus lead to pancreatic β-cell damage and increased insulin resistance. The active component, TQ, helps reduce oxidative stress and protect β-cell integrity, reducing insulin resistance. TQ may also reduce the expression of gluconeogenic enzymes and intestinal glucose absorption [86,87].

An analysis of several clinical trials has highlighted the effects of NS and its active component TQ on patients diagnosed with T2DM and other associated conditions. Thus, 17 clinical trials were reviewed, of which only 13 were randomized controlled trials (RCT). Thus, it could be observed that in most of the studies, the levels of fasting blood glucose (FBG), HbA1c, and insulin resistance (HOMA-IR) decreased compared to placebo groups. The effect of NS and TQ administration was also compared with other antidiabetic drugs (e.g., metformin). It was found that the effects of NS were inferior to metformin in reducing FGB and HbA1c, but there were much better effects in terms of insulin resistance. The duration and dosage of NS and TQ treatment were also analyzed. It was found that high doses and long durations led to improved metabolic parameters. In addition to these effects, it was also observed that NS and TQ had effects in reducing inflammatory markers and liver enzymes, and the lipid profile of the patients with non-alcoholic fatty liver disease (NAFLD) was much better [88].

Worryingly, the incidence of patients with metabolic syndrome and NAFLD is increasing worldwide. Fat accumulation in liver cells can lead to inflammation, liver damage, and, in severe cases, cirrhosis and hepatocellular carcinoma. At the moment, there are no drugs that completely treat metabolic syndrome, and those that do exist have adverse effects. In this sense, NS has started to be studied to improve and treat metabolic syndrome. Thus, it was observed that the administration of NS at different doses (400, 200, and 100 mg/kg) inhibited fat accumulation and favored adipocyte hypertrophy, showing results similar to those of metformin. This phenomenon was attributed strictly to the administration of NS without reducing the patients’ food intake. In terms of fat digestion, the administration of NS contributed to the maintenance of zymogen granules, which are responsible for the secretion of pancreatic enzymes, thus reducing fat absorption in the intestine. NS also reduced glycemia, insulin resistance, and HbA1c dose-dependently. NS protected the liver and inhibited the increase in liver weight and service values of enzymes indicative of liver damage (ALT, AST, GGT, LDH, ALP), prevented the accumulation of fat in the liver, and improved the symptoms of NAFLD. NS also protects the kidneys from the development of diabetic nephropathy due to the accumulation of fat, creatinine, and urea levels in the blood. Thus, NS has demonstrated its efficacy in metabolic syndrome, NAFLD, T2DM, and other comorbidities associated with obesity and can be compared with metformin [89].

NS has also been studied for thyroid conditions. For example, a study by Mahdieh Abbasalizad Farhangi et al. [90] explored the effects of NS on thyroid function, serum levels of VEGF and Nesfatin-1, and possible effects on patients diagnosed with Hashimoto’s thyroiditis. The double-blind, placebo-controlled, randomized, double-blind, placebo-controlled clinical trial enrolled 40 patients with Hashimoto’s thyroid, aged 22–50 years, who received either 2 g of NS or a placebo for 8 weeks. Body weight, body mass index, waist circumference, serum levels of TSH, T3, anti-TPO antibodies, VEGF, and Nesfatin-1 were assessed. After NS administration, a significant reduction in body weight, body mass index, and waist circumference was observed. There was also a decrease in TSH and anti-TPO levels and a significant increase in T3 levels. VEGF levels decreased significantly, an important factor in the progression of thyroiditis and cancer risk, while Nesfatin-1 levels did not change. Thus, NS has demonstrated significant benefits in improving thyroid status, confers protection against severe complications (e.g., thyroid carcinoma), and can be used as adjuvant treatment in managing Hashimoto’s thyroiditis.

In a study by Klaudia Ciesielska-Figlon et al. [91], the effect of NS essential oil on the cytokine production capacity and susceptibility to apoptosis of activated T lymphocytes from women with Hashimoto’s thyroiditis was investigated. The study involved nine women with Hashimoto’s thyroiditis and nine healthy women. Parameters such as lymphocyte proliferation, apoptosis, necrosis, and cytokine production (IL-2, IL-4, IL-6, IL-10, TNF, IFN-γ, IL-17A) were analyzed. NS essential oil (at 1:10 dilution) significantly inhibited CD^4+^ and CD^8+^ cell proliferation in both Hashimoto’s thyroiditis and healthy patients. At dilutions of 1:10 and 1:50, the NS essential oil induced apoptosis and necrosis of T lymphocytes in both healthy women and those with Hashimoto’s thyroiditis. NS oil led to a significant decrease in IL-17A cytokines, whereas IL-2 and IL-4 values were increased in healthy patients after administration of NS at dilutions of 1:10 and 1:50, and in Hashimoto’s thyroiditis patients, NS at a 1:50 dilution increased only the levels of IL-4, without affecting IL-2 levels. Following the results obtained, NS may show potential for use in the management of autoimmune diseases, but further tests are needed to validate the results and to understand the detailed mechanisms.

### 4.2. Cardiovascular Benefits

Regardless of socioeconomic class, cardiovascular disease (CVD) remains a leading cause of morbidity and mortality globally. Researchers are looking into plant bioactive substances as supplemental treatments for CVD. Natural products with demonstrated efficacy against several cardiovascular risk factors include *Nigella sativa* and its bioactive components or derived products. These benefits can be attributed to their antioxidant capacity, hypolipidemic, antiatherosclerotic, or antihypertensive properties [92].

Thus, the administration of NS may have beneficial effects for CVD, as it has been observed to reduce ventricular conduction and prevent the adverse effects of isoproterenol (an agent used to induce stress on the heart in experimental studies). NS also reduced inflammation by inhibiting the secretion of pro-inflammatory cytokines, indicating high anti-inflammatory potential. A significant reduction in markers specific to cardiac disease was also observed. For example, CK-Mb (a marker for cardiac muscle damage) levels were considerably reduced after NS administration. In addition, NS is effective in ameliorating myocardial ischemia, with reduced atherosclerotic plaques in the coronary arteries, thus preventing coronary artery disease development. NS affects blood pressure, heart rate, and local inflammation by blocking the pro-inflammatory mediators generated by macrophages. It does this through various pathways, including serotonergic, muscarinic, and adrenergic systems. Studies have shown that NS may work synergically with medications like losartan and amlodipine to improve blood pressure regulation and lower heart rate. However, modifications may be necessary because these interactions affect some medication metabolizing enzymes (CYP3A4 and CYP2C9). However, further studies are still needed to prove the effectiveness of NS against CVD [92,93,94].

### 4.3. Gastrointestinal Health

In traditional Arabic and Islamic medicine, NS has been used over time to treat various gastrointestinal disorders in seed and oil form. Thus, thanks to its NS compounds, it can be used to treat conditions such as hepatic steatosis, hepatitis C, ulcers, *Helicobacter pylori* infections, intestinal inflammation, etc. [95].

Gastric ulcer is a common gastrointestinal disorder characterized by an imbalance between protective factors (e.g., mucus, bicarbonates, endogenous antioxidants) and destructive factors (e.g., infections, high hydrochloric acid production, alcohol consumption). In this context, NS has antioxidant properties that help to reduce free radicals and relieve gastric ulcers. The administration of TQ, the active component of NS, has been shown to reduce inflammation and accelerate ulcer healing. It has also been observed that NS can inhibit ulceration by about 80%, significantly reducing the affected area and gastric acidity and helping to restore the gastric mucosa. NS can also act as an antimicrobial agent against *Helicobacter pylori* (*H. pylori*) infections, which are the main cause of gastric ulcers and cancer. It was observed that the administration of 2 g of NS and omeprazole contributed to the eradication of *H. pylori*, an effect that can be compared with standard therapy with clarithromycin and amoxicillin. Concerning liver diseases, NS produced an improvement in liver enzyme parameters (serum aspartate transaminase (AST), alanine transaminase (ALT)), contributing to a significant reduction in markers of inflammation and weight loss in patients with NAFLD. At the same time, it was observed that NS had beneficial effects in treating hepatitis C, reducing viral load, and improving clinical factors, including red cell count and albumin levels. In intestinal inflammation, TQ is an agonist of peroxisome proliferator-activated receptor-activated receptor-gamma (PPAR-γ), contributing to symptom relief, and indicating the possibility of using TQ as an adjuvant therapy in inflammatory bowel disease. TQ may also induce apoptosis of pancreatic cancer cells by inhibiting proliferation and reducing inflammation. It may also sensitize cancer cells to chemotherapy and reduce drug resistance. The same has been shown in tests on colorectal cancer cells. *Nigella sativa* has broad therapeutic potential due to its anti-inflammatory, antioxidant, and anticarcinogenic properties. However, more clinical studies are needed to establish the optimal dose and fully explore its action mechanisms [95,96].

### 4.4. Respiratory Health

NS is also widely used in treating respiratory and allergic disorders due to its compounds, such as TQ, THQ, and carvacrol, which function as antioxidants, anti-inflammatories, immunomodulators, antihistamines, and bronchodilators. NS has also been shown to act as an important therapeutic agent in certain biological systems by relieving asthma symptoms or other respiratory disorders such as allergic inflammation. NS may relax the smooth muscles of the trachea, which explains the effects of the bronchodilator on lung diseases. In asthma, NS has been shown to reduce inflammation and modulate the immune response, while beneficial effects on lung tissue histology have also been observed. In allergic reactions, NS has been shown experimentally to reduce pro-inflammatory cytokines (e.g., IL-4, IL-5, and IL-13) and cellular inflammation and prevent infiltration of inflammatory cells into lung tissues. In clinical trials, the administration of NS led to a reduction in the symptoms of seasonal allergies, as well as dust mite allergies. It has also been observed that in cases of toxin-induced fibrosis, NS can prevent and significantly reduce oxidative stress and inflammation. These therapeutic effects of NS are associated with its complex mechanisms to inhibit inflammatory mediators and cytokines, the stimulation of β2-adrenergic receptors which contributes to the relaxation of bronchial smooth muscle, and antioxidizing effects leading to the reduction in oxidative stress and protection of lung tissue against toxins and pathogens. Thus, NS could be used as an adjuvant treatment in respiratory and allergic diseases, with promising therapeutic benefits. However, more clinical trials are needed to confirm these large-scale effects [97,98,99].

### 4.5. Role in Cancer Prevention and Treatment

Cancer remains a major disease with an increasing death rate among the world’s population, the second most common cause of death after cardiovascular disease and involving abnormal cell growth that invades other regions of the body. Numerous variables, including genetic abnormalities and epigenetic modifications such as aberrant DNA methylation and histone deacetylation, contribute to the development of this disease. In cancer, many tumor suppressor genes (TSGs) are inactivated by these epigenetic modifications, which contribute to the uncontrolled proliferation of malignant cells. Cancer caused an estimated 10 million deaths in 2020, and in 2018, an estimated 9.6 million people lost their lives to cancer. Cancer is a multifactorial disease, and various factors, such as diet and lifestyle, radiation exposure, and hormonal factors, can contribute to the development of this fatal disease. According to the International Agency for Research on Cancer (IARC), the global incidence of cancer is expected to increase from an estimated 14.1 million new cases in 2012 to an estimated 20.0 million in 2022. At this time, the clinical management of cancer continues to be challenging as conventional treatments such as chemotherapy and radiotherapy have limitations due to their adverse effects and high toxicity profiles. However, over the last five decades, studies have focused on researching and finding new therapeutics, which has led to significant advances in the development of therapeutic options and more in-depth information about different malignancies. Thus, even though research has led to the authorization of numerous treatment strategies (e.g., chemotherapeutic agents, small molecule inhibitors, specific genes or proteins targeting so-called smart drugs, and immunotherapies) that have demonstrated therapeutic potential in treating different forms of cancer, the rate of cancer-related deaths has not changed considerably. Thus, to limit the adverse effects associated with some methods of cancer treatment, studies have focused on evaluating the therapeutic, as well as cancer prevention, potential of herbal derivatives that have been shown to have beneficial effects. NS is one of the plants whose potential against cancer has been intensively studied. Thus, the active components in NS seeds, such as TQ, p-cymene, carvacrol, and quercetin, have proven their antitumor effect in numerous preclinical studies. TQ can intervene in numerous tumorigenic processes and prevent carcinogenesis, tumor growth, invasion, migration, and angiogenesis [100,101,102].

It has been observed that TQ can sensitize some types of tumor cells and make them more susceptible to conventional treatments while also decreasing the adverse effects on normal cells [102]. A study by Salomi et al. [103] demonstrated the selective cytotoxicity effect of TQ on human cancer cells and its increased efficacy in inhibiting tumor growth and metastasis in preclinical studies. Also, another study by Gali-Muhtasib et al. [104] on colon cancer showed that it can slow down tumor growth, and another group of researchers demonstrated the same effect on other cancers, such as breast, stomach, and skin cancer. In two studies by Velho-Pereira et al. [105] and Rajput et al. [106], it was shown that TQ can increase the efficacy of radiotherapy by modulating the cell cycle and inducing apoptosis. It was also observed that TQ prevented radiation-induced metastatic progression of breast cancer. Regarding the interaction with other anticancer drugs, Pazhouhi and Khazaei et al. [107] showed that TQ potentiates the effect of the temozolomide drug in treating glioblastoma by inhibiting the autophagy process, which contributes to tumor resistance. Jafri et al. [108] showed that TQ combined with cisplatin enhanced chemotherapy’s effects on colon and ovarian cancer, reducing drug resistance and improving the cytotoxic effects at subtherapeutic doses. Also, the combination of TQ with 5-fluorouracil reduced the toxic effects on the liver and kidney, demonstrating an excellent safety profile as observed in the study by Kensara et al. [109]. TQ also protected organs from chemotherapy-induced toxicity in several cancer models, such as colon, ovarian, and lung cancer [102]. The antitumoral effects of carvacrol were investigated in a study conducted by Leilei Li et al. [110]. The researchers aimed to highlight the antitumor potential on breast cancer cell lines, and their main findings were as follows: (1) In tests performed on five different cell lines, carvacrol had the best effect on the MDA-MB-231 cell line, while the MCF-7 cell line was the most resistant. (2) At concentrations higher than 300 μM, carvacrol induced apoptosis in all cell lines. (3) Also, carvacrol significantly increased the proportion of cells in the G1/G0 phase and reduced the proportion of cells in the S and G2/M phases, indicating a cell proliferation arrest. (4) Researchers have suggested that carvacrol may affect the TRPM7 ion channel, representing a potential pharmacologic target for carvacrol. However, further research is needed to identify the direct targets of carvacrol. Another study by Ashley B. Ward et al. [111] aimed to highlight the potential of quercetin on prostate cancer cells. Thus, the researchers observed that putting cancer cells in contact with quercetin induced a considerable decrease in cell viability that was time- and dose-dependent, without affecting healthy cells. The decrease in viability was found to be due to apoptosis and necrosis, which occurred as a result of altered mitochondrial integrity and disruption of homeostasis (ROS). Quercetin shows therapeutic potential for treating prostate cancer by inducing selective cell death in tumor cells without affecting normal cells.

Thus, NS seeds could represent an alternative treatment against cancer or be used as an adjuvant therapeutic agent. However, further studies demonstrating the antitumor effect of active compounds of NS are needed for further exploration.

### 4.6. Neuroprotective Effects and Memory Improvement

It has also been observed that TQ may also have anticonvulsant, antidepressant, antianxiety, and antipsychotic effects and could be used to treat drug abuse or addiction or to improve memory and cognitive function. Thanks to its antioxidant effect, TQ may protect brain cells from oxidative stress, which occurs particularly in memory-related regions. Neuroinflammation is one of the main causes of diseases such as Parkinson’s and Alzheimer’s, which can increase ROS production. In this regard, TQ inhibits the increased activation of NF-KP and its binding to DNA, consequently inhibiting neuroinflammation. TQ may protect brain cells from various injuries and inflammation due to its antioxidant properties and anti-inflammatory effects. TQ administration may inhibit the release of mRNA and TNF-α leukotrienes, including IL-6, IL-1β, and prostaglandin synthesis. Also, it has been observed that TQ can inhibit the enzyme nitric oxide synthase (iNOS), which will lead to a decrease in nitrite (NO_2_^−^). Therefore, reducing the expression of the iNOS protein can effectively alleviate inflammatory and autoimmune diseases. TQ also prevents neuronal damage by suppressing TLR receptor activation and inflammatory signaling pathways, decreasing the risk of apoptosis and neurotoxicity. TQ may reduce glutamate toxicity to neurons, and also prevent mitochondrial dysfunction, and reduce ROS production, protecting cells from apoptosis. In Alzheimer’s disease, TQ may reduce neurotoxicity caused by amyloid-beta peptides (Aβ) and prevent the accumulation of amyloid plaques, thereby protecting neurons. In Parkinson’s disease, TQ helps preserve dopaminergic neurons and combats the disease’s motor symptoms by reducing oxidative stress and protecting mitochondria against the MPTP toxin. TQ improves spatial memory by protecting neurons from oxidative stress by eliminating free radicals. Studies have shown that TQ inhibits acetylcholinesterase (AChE), thereby increasing acetylcholine (ACh) levels, essential for long-term memory. TQ prevents memory loss in animals and is comparable to donepezil, an AChE inhibitor. In addition, TQ increases the ability to consolidate and recall information, protecting against memory loss in diabetic animals [3,112,113].

## 5. Clinical Studies and Evidence-Based Applications

The seeds of NS, also known as black cumin, have been used since ancient times and continue to be used in the modern era to treat or relieve a wide variety of conditions (e.g., diabetes, bacterial and viral infections, allergies, respiratory diseases, digestive disorders, inflammatory diseases, cardiovascular diseases, and cancer), as they possess anti-inflammatory, antioxidant, antimicrobial, and immunomodulatory properties [3,10,11,12]. Thus, this section aims to summarize some of the studies that attest to NS’s beneficial effects in treating some of these conditions.

In relation to diabetes mellitus, a study by Mohamad Fawzi et al. [88] explored the efficacy and safety of using NS seeds and the active substance TQ in the management of this chronic disease. The study used the PRISMA guidelines, so they selected 17 clinical trials for analysis. Thus, the analysis targeted diabetic patients, either suffering from type 1 or type 2 diabetes, but also healthy subjects, and the parameters followed were fasting blood glucose (FBG), glycosylated hemoglobin (HbA1c), and insulin resistance (HOMA-IR). It was observed that NS had positive results in terms of reducing fasting blood glucose and HbA1c and improved insulin resistance and pancreatic beta-cell function. In most studies, blood glucose was reduced compared to placebo groups. For example, the study conducted by Kooshki et al. [114] showed a significant decrease in FBG (65.4 mg/dL) in the NS-treated group compared to placebo. At the same time, another study by Kaatabi et al. [115] observed that after 12 months, HbA1c levels decreased by 0.84% in the NS-treated group, while an increase of 0.14% was recorded in the placebo group. Thus, it can be seen that NS and TQ may represent potential strategies for diabetes treatment, although further studies are needed to highlight the mechanisms of action [88].

Obesity is a leading cause of metabolic disorders such as CVD and T2DM. Thus, NS has been investigated for its antioxidant, anti-inflammatory, and metabolism-regulating properties, having numerous active compounds, including flavonoids and alkaloids with pharmacologic potential. The antioxidant activity was evaluated by the ability to scavenge DPPH radicals, while the anti-inflammatory potential was focused on analyses of nitric oxide (NO), prostaglandin E2 (PGE2) production, and pro-inflammatory gene expression, performed on Raw264.7 cells treated with NS extract. The anti-adipogenic effects were determined by observing the lipid accumulation capacity and the expression of adipogenesis-associated genes, such as PPARγ and C/BEPα, in differentiated 3T3-L1 cells. Thus, NS was shown to have a concentration-dependent, increased DPPH uptake capacity without affecting cell viability at concentrations below 30 µg/mL. At the same time, administering 30 µg/mL of NS led to the significant inhibition of PGE2 and NO production in LPS-simulated cells, preventing the expression of the inflammatory mediators TNF-α and IL-6. The effect was supported by inhibition of phosphorylation of molecules involved in the inflammatory response, such as NF-κB and MAPK. Regarding the anti-adipogenic effects, it was observed that in 3T3-L1 cells, NS reduced lipid accumulation and the expression of adipogenesis-associated genes, suggesting a potential for reducing excessive adipose differentiation. The study emphasized the potential of black cumin extract as an anti-inflammatory and anti-adipogenic agent, indicating a possible use in herbal formulations for the management of metabolic disorders [116].

Another study by Yuxiao He et al. [117] highlighted the efficacy of NS in treating or relieving the symptoms of allergic rhinitis, which may affect 10–20% of the global population. Thus, the authors conducted a meta-analysis based on randomized clinical trials involving patients of all ages and genders, intervening with NS as monotherapy, either used together with other types of drugs (e.g., loratadine, mometasone, montelukast), which were compared with control groups receiving only conventional treatments. Thus, eight studies were selected that demonstrated an increased efficacy in reducing allergic rhinitis symptoms compared to the control group. In addition, symptoms such as nasal congestion, itching, and sneezing were significantly reduced. In addition to the beneficial effect of NS, minor adverse effects, such as nasal dryness and itching, were also noted, but they were temporary and statistically insignificant. However, the authors emphasized that although the studies provided evidence for the efficacy of NS against allergic rhinitis, there is still a need for larger, well-designed studies [117].

Another study [74] focused on MRSA infections, a major public health problem, especially due to the resistance of this pathogen to multiple antibiotics. Thus, the antimicrobial potential of NS was tested in vitro and in vivo to treat MRSA infections. In the in vitro tests, NS was applied to MRSA strains harvested by humans and animals to determine the ability of NS to inhibit bacterial growth. In vivo tests were performed on 200 inbred rabbits divided into four groups. Different concentrations of NS (125, 250, 500 mg/kg) were administered to each group to observe the effects on immunity and intestinal integrity. Regarding the antimicrobial effect, NS administration had an effect on MRSA strains of animal (77.8%) and human (64.3%) origin. At the same time, NS led to the development of antibiotic susceptibility of the strains, indicating a synergistic activity of NS with antibiotics. Also, rabbits fed NS showed an increase in the expression of genes supporting the integrity of the intestinal barrier and genes involved in the immune response. Higher levels of NSE increased the expression of tight junction proteins and molecules involved in the integrity of the intestinal epithelium, and NSE reduced inflammatory markers such as IL-6 and TNF-α [74].

On the other hand, another study conducted by Khalid A. Bin Abdulrahman et al. [118] aimed to highlight the antiviral potential of NS seeds against SARS-CoV-2. Thus, this analysis was performed on a sample of 262 patients diagnosed with COVID-19, without severe symptoms, who were randomly divided into seven groups. Six groups received varying doses of NS seeds (powder or whole seeds), while the placebo group received activated charcoal capsules, which were administered twice daily for ten days. Symptoms were monitored, and inflammatory markers and CD cell profiles were measured before and after treatment. The results showed that there were no significant differences between groups in clinical symptoms (e.g., wheezing, cough, loss of sense of smell). Also, white blood cell (WBC) levels significantly increased post-treatment, and most inflammatory markers had no significant differences between groups. Also, some cellular subtypes (CD3, CD4, CD8) showed slight changes in some groups but without a consistent pattern, suggesting a clear immunomodulatory effect of NS in the studied range. The authors concluded that treatment with NS for 10 days had no significant impact on symptoms or inflammatory markers in patients diagnosed with COVID-19. At the same time, they suggested that NS might be more effective in long-term treatments, especially in chronic or severe conditions, but further tests are needed [118].

A study by Parisa Shoaei-Hagh et al. [119] highlighted the potential of NS oil in reducing the risk of cardiovascular disease in hypertensive patients. Thus, a double-blind clinical trial was conducted involving 55 hypertensive patients who were divided into two groups. Patients in the first group were given 2.5 mL of NS oil, while patients in the second group were given 2.5 mL of sunflower oil (placebo) for 8 weeks. Thus, the main objectives were to measure blood pressure (systolic and diastolic), cholesterol levels, low- and high-density lipoproteins (LDL and HDL), blood glucose, and some markers of oxidative stress (malondialdehyde—MDA and glutathione reductase—GR). A significant reduction in blood pressure, LDL, cholesterol levels, and blood glucose was observed compared to the placebo groups. There was also an increase in HDL levels and a reduction in oxidative stress, with no adverse effects reported.

**Figure 4:**
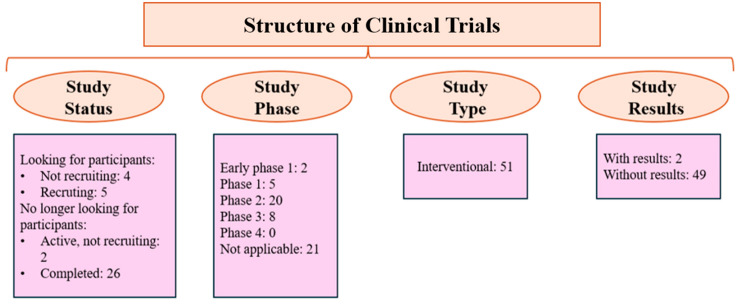
The structure of clinical studies available on ClinicalTrials.gov as of October 2024.

According to the ClinicalTrials.gov platform, several studies might bring new promising results, as 51 clinical trials have been identified as of October 2024 (Figure 4). The therapeutic effect of NS has also been studied in other clinical trials, as illustrated in Table 2.

The mentioned studies have highlighted the potential of NS to be a therapeutic agent that can be used both as monotherapy and as an adjuvant in different diseases. However, it is worth mentioning that further studies are currently needed, as there are only a few existing ones, and their design should be varied to better understand the mechanism of action of NS in the given contexts. It has also been highlighted that NS could be more efficient in long-term treatments for chronic or severe conditions, and one of the studies even found that it was limited by the 10-day duration of NS treatment [74,88,116,117,118,119].

## 6. Pharmacokinetics and Dosage

Thymoquinone (TQ) is the active component of NS seeds and is responsible for most therapeutic effects. It has been observed that pharmacokinetic behavior is gender-dependent, and this may be due to parameters such as body weight, adipose tissue distribution, plasma protein binding, enzyme metabolization, and drug transport activities. In mouse model studies, TQ was well tolerated at concentrations of 5 mg/kg administered intravenously (IV) and 20 mg/kg administered orally (PO). It was also observed that TQ is rapidly eliminated from plasma after IV administration. With oral administration, it could be seen that TQ is slowly absorbed, compared to its rate of distribution, and rapidly eliminated. The bioavailability of TQ was estimated to be 58% [60,134,135]. TQ can be delivered to improve bioavailability by utilizing known nanoparticles and nanostructured lipid scavengers that improve drug reactivity. For example, TQ can be encapsulated using hydrophilic biodegradable polymers (e.g., polyethylene glycol (PEG)), which can improve solubility and systemic bioavailability. The pharmacokinetic properties of thymoquinone were also studied, and it was observed that its encapsulation in lipid-based nanoparticles (TQ-NLC) led to a much higher bioavailability of the active substance. Thus, when administered orally, absorption occurs in the intestine. It is assumed that TQ-NLC is taken up by the gastrointestinal tract and moved to the reticuloendothelial organs. At the same time, thymoquinone-loaded NLCs undergo lipid digestion so that the triglycerides constituting the NLCs are broken down into monoglycerides and free fatty acids by pancreatic and duodenal enzymes, which release the active substance into the intestines. It could be observed that TQ-NLC was less absorbed in the liver compared to the intestines two hours after administration, which is explained by the lymphatic uptake of TQ-NLC in the passage of the gastrointestinal tract. After absorption, long-chain fatty acids or lipids are fused into chylomicrons, which are then absorbed into the intestinal lymph. Since the size of the chylomicrons is usually large (about 80 nm), they enter the lymphatic system instead of the blood capillaries, bypassing the first metabolism of the drug associated with them. After intravenous administration, TQ-NLCs have been observed in the kidney, liver, and spleen, and this is due to macrophages reaching these tissues as residues. Thus, it was observed that TQ-NLC has access to multiple tissues and organs, generating the first concentration peak, after which TQ-NLC returns to the plasma and is further redistributed. This is explained by the fact that there is reabsorption in the intestinal tract due to enterohepatic recirculation, which leads to reabsorption and recycling in tissues and two-site absorption (in the stomach and intestine), and the enterohepatic component includes the gastrointestinal tract and kidneys. Thus, it was observed that the TQ-NLC system exhibited a slow and sustained release of TQ. TQ-NLC was better absorbed following IV administration compared to PO administration, although better bioavailability was demonstrated for the latter method of administration [136]. In a cancer study, PEG-TQ nanoparticles prevented cancer cell migration and induced breast cancer apoptosis in vivo. In nano-thymoquinone form, it was more effective than thymoquinone in inhibiting the proliferation of human mammary adenocarcinoma MCF-7 cells in a concentration-dependent manner. Also, the combination of thymoquinone with conventional anticancer drugs may improve the chemotherapeutic potential. It has been shown that it may enhance the effects of doxorubicin, demonstrating high efficacy against MCF-7 cells. Also, the use of thymoquinone and tamoxifen on the viability of estrogen-negative MDA-MB-231 and MCF-7 human breast cancer MDA-MB-231 and MCF-7 led to the observation of a synergistic effect, as together, they led to decreased cell viability and induced apoptosis in both cell lines. TQ, together with paclitaxel (an antitumor agent used in triple-negative breast cancer), inhibited cancer growth in cell culture and induced the apoptosis of Brca1, p21, and Hic1 tumors [137].

Depending on the desired therapeutic effect, NS may have different dosages and effects that have been tracked. For example, the administration of 1.5 to 3 mL/day of NS oil for 20 days in diabetic patients led to a significant decrease in glycated hemoglobin A1c and random blood glucose concentrations [138]. Also, the administration of NS seeds (1–3 g/day) to T2DM patients had the following effects. The administration of 1 g/day of seeds led to an increase in high-density lipoprotein cholesterol (HDL-c) levels after 3 months, whereas two and three g/day of *N. sativa* seeds significantly decreased serum total cholesterol (TC) and triglyceride (TG) levels, as well as low-density lipoprotein cholesterol (LDL-c) and increased plasma HDL-c [139]. In comparison to the corresponding baseline values and the control group, it was found that a one-year course of 2 g daily of black cumin resulted in a significant decrease in systolic, diastolic, and mean arterial blood pressure, heart rate, TC, LDL-c, the fractions of TC/HDL-c, and LDL-c/HDL-c, while serum HDL-c was suggestively elevated [140]. Moreover, it was observed that a dosage of 2 g/day of NS seeds had a clinically valuable anti-*H. pylori* effect, and it was suggested that NS can be used as a potential treatment for *H. pylori*-induced gastric ulcers [141].

In general, the bioactive components in NS seeds show low toxicity and have a wide safety margin [13]. A review of several animal studies on the toxicity of NS seeds yielded the following results. PO and IV at doses of 10, 15, 20, 25, 25, 30, 40, and 50 mL/kg, 0.25, 0.5, 1, 2, 3, 4, and 6 mL/kg, respectively, were administered to male and female mice. When administered orally or intraperitoneally, NS caused behavioral abnormalities and restlessness at all doses, which were followed by somnolence. In addition, all mice died 12 h after the oral administration of 50 mL/kg NS or 4 mL/kg intraperitoneally [142]. Male Sprague Dawley rats were given an oral 10 mL/kg aqueous extract of NS seeds for 14 days to investigate the subacute toxicity of caraway seeds. The aqueous extract used in this investigation was not described. Although serum alkaline phosphatase (ALP) levels stayed unchanged and histopathologic examinations revealed no impairment, the results showed a significant increase in gamma-glutamyl transferase (γ-GT) amounts; perhaps this was due to an anesthetic effect on ALP release. Rats and mice given 10 mL/kg NS seed oil orally for 48 h did not die or exhibit obvious toxicity [143]. To test the subchronic toxicity of NS on animals, ground NS seeds were administered at concentrations of 20 and 100 g/kg to 7-day-old Hibro broiler chicks for 7 weeks. It was observed that these concentrations of NS damaged the liver, as evidenced by increased AST (serum aspartate transaminase) and ALT (alanine transaminase) values, but also by decreased serum albumin and cholesterol levels. A total of 100 g/kg NS led to a significant reduction in red blood cell and hemoglobin levels [144]. To evaluate the chronic toxicity of NS, 2 mL/kg body weight NS-fixed oil was administrated to Wistar-Kyoto rats for 12 weeks. It was observed that the levels of leukocytes and platelets significantly decreased, while the hematocrit, hemoglobin, and mean corpuscular hemoglobin concentrations were significantly increased. There were no signs of histopathological damage in the heart, kidneys, liver, or pancreas. The researchers concluded that the NS-fixed oil has a wide margin of safety at therapeutic doses, but more studies are needed for the hematological impact [142].

It was observed that administering 5 mL/day of NS to volunteers for 26 days did not cause any adverse effects at the hepatic, renal, or gastrointestinal level. Also, the administration of 3g/day for 3 months in obese patients did not lead to adverse effects. Both the oil and the crushed NS seeds led to significantly higher γ-GT and ALP activity, and the usage of total oil was linked to a notable increase in blood levels of AST and ALT. CYP2D6 and CYP3A4 in human liver microsomes and healthy human volunteers were inhibited by 5 g/day of NS seeds [145,146,147]. In this regard, NS is safe, and human clinical trials have not shown any noticeable adverse effects from taking NS. The use of NS in all biological systems is thought to be relatively risk-free because of the large amount of research performed to ascertain its safety. NS is acceptable to use in food and medicine in small amounts for brief periods. However, more detailed investigations are still needed to have a clearer insight into the toxicological profile of NS [13,148].

## 7. Conclusions

*Nigella sativa* (NS) is a potential herbal treatment for various conditions, such as gastrointestinal, inflammatory, infectious, immunomodulatory, respiratory, cardiovascular, cancer, and diabetes. NS seeds contain phytochemical compounds such as thymoquinone, p-cymene, α-thujene, longifolene, β-pinene, α-pinene, and carvacrol, which have important biological properties that make them exploitable by researchers for the discovery and innovation of revolutionary treatments in the medical field. NS seeds can synergize with other drugs, such as chemotherapeutic agents or antibiotics, reducing the required therapeutic dose, improving treatment outcomes, and aiding in overcoming obstacles such as drug resistance. As highlighted by the overview of the relevant research studies discussed in this paper, NS has the potential to be a therapeutic agent both as a monotherapy and as an adjuvant. Although many studies have proved the therapeutic properties of NS, more in-depth research is required to better understand the mechanism of action of NS in the given contexts. Moreover, NS is acceptable to be used in food and drug manufacturing but in small amounts and to be administered for brief periods. Therefore, to be eligible for use in therapies, NS needs more detailed investigations to obtain clearer insights into the toxicological profile of NS and verify if it can be safely used in higher quantities and for longer times. To conclude, the findings reviewed in this paper offer an updated framework for future research in the field, encouraging further in-depth interdisciplinary studies that would help advance present NS-based therapeutic strategies to reach clinical settings.

## Figures and Tables

**Table 1:** *Nigella sativa* bioactive compounds and their biological effects.

Bioactive Compounds	Plant Part	Biological Effects	References
Thymoquinone	Seeds	Hepatoprotective Anti-inflammatory Antioxidant Antitumoral Antimicrobial Antihistamine Immunomodulator Neuroprotective Gastroprotective Anti-nociceptive	[19,20,21,22,23,24]
p-Cymene	Seeds	Antioxidant Anti-inflammatory Antimicrobial Antitumoral Antiparasitic Antidiabetic Antiviral Antifungal Anti-nociceptive Immunomodulatory Vasorelaxant Neuroprotective Analgesic	[19,20,25,26]
α-Pinene and β-Pinene	Seeds	Antibiotic resistance modulator Anticoagulant Antitumoral Antimicrobial	[19,20,27]
Limonene	Seeds	Antitumoral Antiviral Anti-inflammatory Antimicrobial Antidiabetic Antinociceptive Antioxidant Analgesic Neuroprotective	[19,20,28,29,30]
Longifolene	Seeds	Antibacterial Antitubercular Anticoagulant Antitumoral	[19,20,31]
α-Terpineol	Seeds	Antioxidant Antitumoral Anticonvulsant Antiulcer Antihypertensive Anti-nociceptive Antioxidant Anti-inflammatory Analgesic Neuroprotective Anti-diarrheal	[19,20,32,33]
Carvone	Seeds	Antidiabetic Anti-inflammatory Antitumoral Neuroprotective Antimicrobial Antiarthritic Anticonvulsant Immunomodulator	[19,20,34,35]
Carvacrol	Seeds	Antimicrobial Anti-inflammatory Antioxidant Antitumoral Antiparasitic Analgesic Hepatoprotective	[19,20,34,36,37,38,39]
T-anethol	Seeds	Anti-metastatic Antioxidant Antimicrobial Antiviral Anti-inflammatory Chemopreventive Antitumoral Antidiabetic Neuroprotective Antithrombotic	[19,20,40,41]
Gallic acid	Roots Shoots	Antioxidant Antibacterial Antitumoral Antiviral Antihistaminic Anti-inflammatory Anti-angiogenic	[19,20,42,43,44,45]
Kaempferol	Seeds	Antioxidant Antibacterial Anti-inflammatory Antiviral Neuroprotective Analgesic Antitumoral Antihistaminic Antidiabetic Neuroprotective	[19,20,46,47]
Quercetin	Roots Shoots	Antioxidant Anti-inflammatory Antibacterial Antiviral Vasodilator Antitumoral Gastroprotective Immunomodulator	[19,20,21,42,48,49]
α-Hederin	Seeds	Anti-inflammatory Antiviral Antioxidant	[19,20,50]

**Table 2:** Summary of clinical studies about *Nigella sativa* as a therapeutic agent.

ClinicalTrials.gov ID	Official Title	Conditions	Intervention/Treatment	Study Type	Phase	Ref.
NCT00327054	Effectiveness of *Nigella sativa* (Kalonji) Seed in Dyslipidemia: A Randomized Controlled Trial	Hypercholesterolemia Hypertension Diabetes Mellitus Metabolic Syndrome X	*Nigella sativa* seed	Interventional	Phase 1 Phase 2	[120]
NCT03959306	Effect of Black Seed Oil on Markers of Endothelial Dysfunction in Patients With Type 2 Diabetes Mellitus	Diabetes Mellitus, Type 2	Dietary Supplement: Black Seed Oil Drug: Anti-Diabetics	Interventional	Phase 2	[121]
NCT03776448	The Effect of 2 Grams Daily Supplementation of Thymoquinone-Containing Sativa Nigra Oil on Blood Glucose Levels of Adults: A Placebo-controlled Double-blinded Randomized Controlled Trial	Diabetes Mellitus	Dietary Supplement: Sativa Nigra oil Softgels Dietary Supplement: Activated Charcoal Softgels	Interventional	N/A	[122]
NCT05343767	A Phase II, Randomized, Double-Blind, Double Dummy, Active-Controlled Clinical Trial to Investigate the Efficacy and Safety of Low-Glu in Patients Newly Diagnosed With Type II Diabetes Mellitus	Type2 Diabetes Mellitus	Dietary Supplement: Natural Wellness Low-Glu low dose Dietary Supplement: Natural Wellness Low-Glu high dose Other: Metformin	Interventional	N/A	[123]
NCT06140485	A Phase III, Randomized, Double-Blind, Double-Dummy, Active- Controlled Clinical Trial to Investigate the Efficacy and Safety of NW Low-Glu^®^ in Patients With Type II Diabetes Mellitus	Diabetes Mellitus, Type 2	Dietary Supplement: NW Low-Glu^®^ Drug: Metformin	Interventional	Phase 3	[124]
NCT05450289	The Efficacy of *Nigella sativa* in Children With House Dust Mite-Induced Respiratory Allergy Receiving Immunotherapy	House Dust Mite Allergy	Drug: *Nigella sativa* Oil Drug: Allergen Specific Immunotherapy Drug: Standard pharmacotherapy	Interventional	N/A	[125]
NCT04347382	The Role of Honey and *Nigella sativa* in the Management of COVID-19: A Randomized Controlled, Open-label, Add-on Trial in Pakistan	Coronavirus Infection Sars-CoV2	Drug: Honey Drug: *Nigella sativa*/Black Cumin Drug: Placebos	Interventional	Phase 3	[126]
NCT04401202	Effects of *Nigella sativa* as a Treatment of Patients With Upper Respiratory Tract Infection Caused by SARS-coronavirus-2: a Prospective, Randomized, Open-label, Controlled Clinical Study	Coronavirus Infection Sars-CoV2	Dietary Supplement: *Nigella sativa* oil	Interventional	Phase 2	[127]
NCT05952102	Role of *Nigella sativa* Oil as an Adjuvant Therapy in the Treatment of Pediatric Pneumonia	Pneumonia	Drug: *Nigella sativa* Oil capsule	Interventional	N/A	[128]
NCT05494164	Effect of *Nigella sativa* on Selected Outcomes Among Patients With Chronic Rhinosinusitis: Prospective Clinical Trial	Chronic Rhinosinusitis	Other: *Nigella sativa* nasal oil drops Drug: standard treatment	Interventional	N/A	[129]
NCT04914767	The Effectiveness of *Nigella sativa* in the Treatment of SARS COV2 (COVID-19)	SARS-CoV2 Infection	Drug: *Nigella sativa* Drug: Placebo	Interventional	Phase 1	[130]
NCT04914377	A Randomized, Double-Blind, Placebo-Controlled Study to Evaluate the Safety and Efficacy of TQ Formula in Treating Participants Who Have Tested Positive for Novel Coronavirus 2019 (BOSS-COVID-19)	COVID19	Drug: TQ Formula/Tab	Interventional	Phase 2	[131]
NCT05069246	The Clinical Assessment of *Nigella sativa* Oil vs. Chlorohexidine as a Therapeutic Aid for Gingivitis, Effect on Gingival IL-6 and IL-18, and Antimicrobial Efficacy.	Gingivitis	Other: *Nigella sativa* oil Drug: Chlorhexidine mouthwash	Interventional	Phase 2	[132]
NCT04553705	Impact of Different Treatment Modalities on Immunity Against COVID-19	COVID-19 Immunodeficiency	Drug: Omega 3/*Nigella sativa* Oil Drug: Omega 3/*Nigella sativa* Oil/Indian Costus Drug: Omega 3/*Nigella sativa* Oil/Quinine pills Drug: Omega 3/*Nigella sativa* Oil/Anise seed capsule Drug: Omega 3/*Nigella sativa* Oil/Deglycyrrhizinated Licorice Drug: Active Comparator	Interventional	Phase 2 Phase 3	[133]

N/A—Not Applicable.

## Data Availability

Not applicable.
